# The Daily Practice of Pancreatic Enzyme Replacement Therapy After Pancreatic Surgery: a Northern European Survey: Enzyme Replacement After Surgery

**DOI:** 10.1007/s11605-012-1927-1

**Published:** 2012-06-19

**Authors:** Edmée C. M. Sikkens, Djuna L. Cahen, Casper van Eijck, Ernst J. Kuipers, Marco J. Bruno

**Affiliations:** 1 Department of Gastroenterology and Hepatology, Erasmus University Medical Center, PO Box 2040, 3015 CE Rotterdam, The Netherlands; 2 Department of Surgery, Erasmus University Medical Center, Rotterdam, The Netherlands

**Keywords:** Chronic pancreatitis, Pancreatic cancer, Exocrine pancreatic insufficiency, Pancreatic surgery, Pancreatic enzyme replacement

## Abstract

**Introduction:**

After pancreatic surgery, up to 80 % of patients will develop exocrine insufficiency. For enzyme supplementation to be effective, prescribing an adequate dose of pancreatic enzymes is mandatory but challenging because the required dose varies. Data on the practice of enzyme replacement therapy after surgery are lacking, and therefore, we conducted this analysis.

**Methods:**

An anonymous survey was distributed to members of the Dutch and German patient associations for pancreatic disorders. The target population consisted of patients with chronic pancreatitis or pancreatic cancer who had undergone pancreatic surgery and were using enzymes to treat exocrine insufficiency. Results were compared to a similar group of non-operated patients.

**Results:**

Ninety-one cases were analyzed (84 % underwent a resection procedure). The median daily enzyme dose was 6, and 25 % took three or less capsules. Despite treatment, 68 % of patients reported steatorrhea-related symptoms, 48 % adhered to a non-indicated dietary fat restriction, and only 33 % had visited a dietician. The outcome was equally poor for the 91 non-operated patients.

**Conclusion:**

Most patients suffering from exocrine insufficiency after pancreatic surgery are undertreated. To improve efficacy, physicians should be more focused on treating exocrine insufficiency and educate patients to adjust the dose according to symptoms and their diet.

## Introduction

In exocrine insufficiency, the pancreas is unable to deliver sufficient quantities of digestive enzymes to the small intestine. Because of the large residual capacity of the pancreas, exocrine insufficiency becomes clinically apparent only when 90 % of the function is lost.^1^,^2^ After pancreatic surgery, the exocrine function is determined by the underlying pancreatic disease and the chosen surgical procedure (depending on the extent of the resection and the restoration of gastrointestinal tract continuity).^3^,^4^ Postoperatively, exocrine insufficiency is observed in 65 to 80 % of the patients with chronic pancreatitis.^5^,^6^ In patients with pancreatic cancer, exocrine insufficiency is found in 68 to 92 % before surgery and in 80 % after surgery.^7^

Exocrine insufficiency leads to malabsorption, which causes steatorrhea, weight loss, and malnutrition.^8^–^11^ It is associated with deficiencies of the fat-soluble vitamins (vitamin A, D, E, and K), magnesium, calcium, and essential fatty and amino acids.^12^–^15^ To reduce morbidity and mortality, patients should be treated with a sufficient dose of pancreatic enzymes.^16^–^19^ In clinical practice, enzyme replacement therapy proves challenging because the optimal enzyme dose is highly variable, depending on the remaining pancreatic function, the postsurgical anatomy, and the dietary fat content. Unfortunately, physicians are often not well informed about the need to supplement exocrine insufficiency in a flexible and patient-tailored manner. In addition, exocrine insufficiency is frequently overlooked because the primary attention is directed towards treatment of the underlying disease. This may be especially true for surgeons who are confronted with a patient with pancreatic cancer because their main concern is to explore curative options.

Remarkably, data on the clinical practice of enzyme replacement therapy in surgically treated patients are lacking. Therefore, this survey was conducted to evaluate the daily practice of enzyme replacement therapy in postoperative patients.

## Methods

This prospective, cross-sectional study was approved by the Medical Ethics Committee of the Erasmus University Medical Center in Rotterdam. In 2010, an anonymous survey was distributed among the members of the Dutch and German patient associations for pancreatic disorders. The target population consisted of surgically treated patients with chronic pancreatitis or pancreatic cancer who were using enzymes to treat exocrine insufficiency. We also assessed the responding members who fulfilled the criteria of the target population, but had not been operated upon.

The survey was distributed by mail and had to be returned within 3 months. After 4 weeks, a reminder was sent out to the members that had not responded. The survey contained free field and multiple-choice questions and took about 10 min to complete. The items evaluated in the survey were the daily enzyme dose (recalculated as the number of capsules containing 25,000 FIP (Fidiration Internationale Pharmaceutique) units of lipase), the timing of enzyme ingestion, the presence of steatorrhea-related symptoms (bloating; abdominal cramps; bulky, sticky, and fatty stools), referral to a dietician, and restrictions in fat intake (either recommended by a physician or dietician, or self-imposed).

Statistical analyses were conducted using SPSS 17.0. Results were expressed as medians and percentiles using the appropriate *t* tests or nonparametric tests for continuous variables. For categorical data, the *Χ*² test was used.

## Results

Two hundred and sixty-five members responded to the survey, of which 182 (69 %) had chronic pancreatitis or pancreatic cancer and were using pancreatic enzymes for exocrine insufficiency. The baseline characteristics of these 182 patients are described in Table 1. Ninety-one of these patients had undergone pancreatic surgery (84 % underwent a resection procedure and 16 % a drainage procedure; Table 2). The remaining 91 patients had not been operated upon.

**Table 1:** Baseline characteristics of 182 operated and non-operated patients with exocrine insufficiency caused by chronic pancreatitis and pancreatic cancer

	Operated patients *N* = 91	Non-operated patients *N* = 91
Age—median (IQR)	60 (49–68)	57 (50–67)
Male sex—*n* (%)	44 (48)	41 (45)
Chronic pancreatitis—*n* (%)	54 (59)	83 (91)
Pancreatic cancer—*n* (%)	37 (41)	8 (9)
Supplementation duration—median mo (IQR)	54 (28–131)	60 (49–68)

**Table 2:** Type of surgical procedure in the 91 operated patients with chronic pancreatitis and pancreatic cancer

	Chronic pancreatitis *N* = 54	Pancreatic cancer *N* = 37
Type of surgical procedures		
Whipple—*n* (%)	20 (37)	31 (84)
Distal pancreatectomy—*n* (%)	5 (9)	3 (8)
Pancreatic head resection—*n* (%)	14 (26)	1 (3)
Total pancreatectomy—*n* (%)	–	2 (5)
Type of drainage procedures		
Pancreaticojejunostomy—*n* (%)	8 (15)	–
Other drainage procedures—*n* (%)	7 (13)	–

The enzyme preparations used by the study population were all enteric coated and of porcine origin, Creon in 52 % (Abbott, Solvay Pharma), Panzytrat in 32 % (Tramedico, Aptalis Pharma), and Pancrease in 16 % (Janssen-Cilag). Eighty-five percent used a high-dose preparation (25,000 FIP units of lipase). Twenty-four patients (26 %) took the enzymes before their meal, 62 (68 %) during a meal, and 11 (12 %) after a meal. Forty-nine patients (54 %) reported to take additional enzymes when they had a snack.

After surgery, the reported median enzyme dose was six capsules per day, and 23 patients (25 %) took three or less capsules per day (Table 3). There was no difference in dosing between the subgroups of patients who had undergone a pancreatic resection and a drainage procedure. Despite treatment, 62 cases (68 %) reported steatorrhea-related complaints, and 36 (39 %) suffered from weight loss. From the patients who took three or less capsules per day, 16 (70 %) suffered from steatorrhea and 11 (48 %) from weight loss, versus 46 (51 %) and 23 (34 %) of the patients who used more than three capsules per day (*p* value 0.769 and 0.219, respectively). Only 33 patients (36 %) reported to have visited a dietician for their exocrine insufficiency, and 44 patients (48 %) kept an unnecessary dietary fat restriction, either prescribed by a physician or dietician, or self-imposed. All these results were comparable to the results of the non-operated patients.

**Table 3:** Treatment outcome of enzyme replacement therapy in 91 surgically treated patients with chronic pancreatitis and pancreatic cancer and 91 non-operated patients

	Operated patients			Non-operated patients
	All *N* = 91	Chronic pancreatitis *N* = 54 (59 %)	Pancreatic cancer *N* = 37 (41 %)	All *N* = 91
Enzyme dose/day^a^; 25 % of patients	≤3	≤4	≤3	≤3
50 % of patients	≤6	≤6	≤5	≤4
75 % of patients	≤8	≤8	≤7	≤6
Symptoms of steatorrhea—*n* (%)	62 (68)	37 (69)	25 (68)	61 (67)
Weight loss—*n* (%)	36 (39)	19 (35)	17 (46)	35 (39)
Fat restriction—*n* (%)	44 (48)	28 (52)	16 (43)	52 (57)
Referral to dietician—*n* (%)	33 (36)	14 (26)	19 (51)	21 (23)

^a^Number of enteric-coated capsules containing 25,000 FIP units of lipase

## Discussion

This study is the first to capture how postoperative patients with exocrine insufficiency are being treated and gives an insight in the clinical practice of pancreatic enzyme supplementation. The results imply that, even in countries with a well-organized health care system, the majority of patients are undertreated.

Although steatorrhea-related symptoms may correlate poorly with the objective measure of steatorrhea, we believe we have gathered some valid data. Despite enzyme replacement, most patients were still bothered by steatorrhea-related complaints, although studies have shown that adequate enzyme therapy can relieve these symptoms completely. Furthermore, guidelines stress that with the proper amount of enzymes, patients should be able to maintain a normal diet. In our study population, most patients were not aware of this and tended to avoid fat. Interestingly, results were equally poor for non-operated patients.

Apparently, surgeons as well as gastroenterologists fail to achieve a satisfactory treatment response in many of their patients. This poor outcome may be explained by the particular intricacies of pancreatic enzyme replacement therapy. The enzyme dose needs to be individually adjusted based on the residual pancreatic function and the dietary fat intake.^20^–^27^ In addition, after surgery, the altered anatomy should be taken into account. This variability in individual dosage response and the lack of practice guidelines make it difficult for physicians to prescribe the proper dose. Also, publications on this subject are scarce, and therefore, physicians are not well informed.

Furthermore, besides theoretical knowledge, effective treatment requires ample diligence of the treating physician to train patients in flexible dosing. However, enzyme supplementation may not seem a major concern in patients with chronic pancreatitis who are treated for severe pain. Moreover, in case of a life-threatening condition such as pancreatic cancer, treatment of exocrine insufficiency may seem futile. Yet, even in these patients, steatorrhea-related symptoms and associated weight loss can be a tremendous burden. A randomized trial by Bruno et al. proved that treatment of exocrine insufficiency is beneficial in such patients. Twenty-one patients with exocrine insufficiency were treated for 8 weeks with either six capsules of a high-dose pancreatic enzyme preparation per day, or a placebo.^16^ All patients received dietary consultation. A significant difference in body weight was observed; patients on pancreatic enzymes gained 1.2 % (0.7 kg), whereas patients on placebo lost 3.7 % (2.2 kg). In addition, the fat absorption coefficient in patients on pancreatic enzymes improved by 12 %, whereas in placebo patients, it dropped by 8 %. Also, guidelines published in Gut have recommended that pancreatic enzymes should be used in exocrine-insufficient patients with pancreatic cancer to maintain weight and increase their quality of life.^28^

The anatomical changes resulting from pancreatic surgery require careful consideration in the diagnosis and treatment of maldigestion.^29^ First of all, the extent of the pancreatic resection is an important denominator for the development of exocrine insufficiency. In addition, the normal digestive physiology is altered in case of reconstructive surgery after a Whipple’s procedure. This may lead to a reduction in exocrine pancreatic secretion due to abnormal postprandial CCK release. Also, asynchrony between gastric emptying and pancreatic secretion in the small intestinal lumen may develop, resulting in improper mixing of enzymes and chyme, a phenomenon called secondary exocrine insufficiency.^30^ Furthermore, changes in gastric and duodenal acidity may influence the denaturation of enzymes and the dissolution of enteric-coated mini-dose units. Finally, after surgery, in particular after a Roux-en-Y anastomosis or pancreaticoduodenectomy, malabsorption may develop due to bacterial overgrowth.

Although studies have shown different gastric-emptying profiles after conventional and pylorus-preserving pancreaticoduodenectomy, long-term results have shown that enzyme replacement therapy with enteric-coated preparations is effective postsurgically.^31^–^33^ Therefore, the pancreatic function should be evaluated postoperatively. A fecal Elastase-1 test should be routinely performed, also in asymptomatic patients, because clinical signs of steatorrhea may be absent as patients tend to limit fat intake to reduce symptoms. Pancreatic enzyme supplementation should be considered in every patient with proven exocrine insufficiency, irrespective of the underlying disease or prognosis. Even in the case of asymptomatic exocrine insufficiency, treatment is indicated because studies have shown that malnutrition may develop in these patients. Moreover, enzymes can also be effective in patients with clinical signs of malabsorption without proven exocrine insufficiency because secondary exocrine insufficiency may be present.

There are very few studies that address how pancreatic enzyme replacement therapy should be dosed. Only one randomized trial has compared the efficacy of a fixed enzyme dosage to self-administration ad libitum according to fat intake in exocrine insufficient patients with chronic pancreatitis.^34^ There was a significant increase from 5 capsules per day during the fixed treatment period to 11 capsules per day during the self-administration period. With this increased dose, a significant decrease in steatorrhea-related symptoms was observed. The authors concluded that efficacy is higher when enzymes are self-dosed by patients in a flexible manner.

Despite the absence of an easy method to establish the adequate dose of pancreatic enzymes, some guidelines can be given. A reasonable starting dose is 50,000 to 75,000 units of lipase for a main meal and 25,000 units for snacks. Subsequently, patients should be instructed to vary the dose according to their dietary fat intake, up to a maximum of 16 capsules a day. They should be explained that the presence of steatorrhea-related symptoms and weight loss imply an insufficient dose of enzymes that needs to be increased. The timing of the capsule ingestion is also important because the enzymes have to be mixed with the chyme to be effective. Patients should be instructed to take the enzymes during or right after a meal.^20^,^35^–^38^ Finally, management of exocrine insufficiency can be greatly facilitated by dietary counseling from a well-trained, specialized dietician. A dietician can teach patients the principles of flexible dosing and prevent them from keeping unnecessary dietary restrictions because many patients tend to avoid fat out of fear for steatorrhea-related complaints.

When treatment response remains unsatisfactorily despite optimal use of enzymes, other steps may be needed. To improve therapeutic efficacy, inhibition of gastric secretion (if gastric acid secretion is preserved) can be attempted by the administration of a proton pump inhibitor. Also, capsules can be opened prior to ingestion, or uncoated enzyme preparations can be prescribed, as accelerated gastric emptying may prevent timely dissolution. If the patient still does not have a satisfactory treatment response, testing for bacterial overgrowth and celiac disease should be considered.

Our study was bound to certain limitations. First of all, due to the design as an anonymous survey, which was required by the medical ethical committee, certain information about the patient population was unavailable. Therefore, we were unable to calculate the response rate, to provide information about the lifestyle of patients, and the etiology and severity of the pancreatitis or pancreatic cancer. Furthermore, data could not be objectified in case of remaining questions or uncertainties. For example, the outcome measure of weight loss could have been influenced by other factors, such as the underlying malignancy. Finally, a selection bias might have occurred. After all, this survey was distributed among members of a patient organization, who are generally highly motivated and better informed. Also, complex pancreatic surgery is mainly executed in tertiary referral centers, where treating physicians are likely to be more experienced in diagnosing and treating exocrine insufficiency. However, if this would be the case, it only stresses our conclusions as results elsewhere are expected to be even worse.

## Conclusion

This is the first study to give insight in the daily practice of pancreatic enzyme usage in patients with exocrine insufficiency after pancreatic surgery. Our results indicate that a substantial proportion of patients are underdosed and that there is ample room for improvement. To accomplish this, physicians should be attentive on treating exocrine insufficiency, also in patients with an unfavorable prognosis. Patients should be better educated in using pancreatic enzymes in a flexible manner, depending on their meal composition. For this, involvement of a well-trained, specialized dietician is desirable.
